# Cough suppression and HRQoL in adult people with cystic fibrosis: an unexplored correlation

**DOI:** 10.1186/s12955-022-02053-2

**Published:** 2022-10-06

**Authors:** Ute Niehammer, Mathis Steindor, Svenja Straßburg, Sivagurunathan Sutharsan, Christian Taube, Matthias Welsner, Raphael Hirtz, Florian Stehling

**Affiliations:** 1 Department of Pulmonary Medicine, Adult Cystic Fibrosis Center, University Hospital Essen - Ruhrlandklinik, University of Duisburg-Essen, Essen, Germany; 2 Paediatric Pulmonology and Sleep Medicine, Children’s Hospital, University of Duisburg-Essen, Hufelandstr. 55, 45147 Essen, Germany; 3 Paediatric Endocrinology, Children’s Hospital, University of Duisburg-Essen, Essen, Germany

**Keywords:** Cystic fibrosis, Adults, Cough suppression, Health-related quality of life, Sex differences, Clinical outcomes

## Abstract

**Background:**

Cough suppression assessed by embarrassment about coughing has been shown in adolescents with cystic fibrosis (CF) and negatively affects health-related quality of life (HRQoL) and clinical indicators of disease severity in adolescent females. However, whether cough suppression exists in adults has been studied as little as its effects on clinical and psychological outcomes beyond adolescence.

**Methods:**

Seventy-one subjects completed the self-reported 'Cystic Fibrosis Questionnaire-Revised (CFQ-R + 14)' and a self-report questionnaire about cough suppression, health-related perspectives, and therapy adherence. The status of CF disease was quantified in terms of the percentage of predicted forced expiratory volume in one second (ppFEV_1_), body mass index (BMI), Pseudomonas aeruginosa, pancreatic status, and CF-related diabetes (CFRD). Additional demographic data for sex, age, graduation, employment, and marital status were assessed.

**Results:**

CS exists in adult CF and is associated with impaired HRQoL but not the overall CF disease status regarding BMI, ppFEV_1_, or health-related perspectives. Despite a higher prevalence of cough suppression in women, no effect of sex regarding either outcome measure was observed.

**Conclusion:**

The results of this study suggest that mental health indicators have an impact on cough suppression.

**Supplementary Information:**

The online version contains supplementary material available at 10.1186/s12955-022-02053-2.

## Introduction

Cystic fibrosis (CF) is the most common genetically inherited disease in the Caucasian population. It is a progressive life-shortening disease that affects several organs and is caused by mutations of the cystic fibrosis transmembrane conductance regulator (CFTR) gene [1]. However, the life expectancy of people with CF (pwCF) has increased continuously over the last few years, which has resulted in a growing population of adult pwCF [2].

Pulmonary involvement continues to be the major cause of morbidity and mortality, with women experiencing a worse course of CF lung disease [3]. Therapy for CF lung disease requires adherence to a strict daily and lifelong treatment regimen. A crucial part of daily CF management is airway clearance techniques (ACTs) to prevent progression of CF lung disease by coughing and expectorating sputum. However, pwCF frequent coughs as a bothersome and stressful symptom. They report being embarrassed by coughing in public situations and having concerns that they might disturb people around them [4]. Furthermore, pwCF feelings of annoyance, irritation, frustration, and worry by frequent coughing [5]. Nonetheless, only a very limited number of studies have reported on cough suppression (CS) in pwCF. In two detailed studies with adolescent pwCF, embarrassment about coughing was used as an indicator of CS [6, 7]. Sex differences were found in a way that girls were more embarrassed about coughing than boys, with the consequence that girls suppressed coughing in public to avoid negative attention. CS has also been discussed as one of the factors contributing to the increased morbidity of female pwCF by affecting therapy adherence. Low adherence to the complex treatment regimens not only influences clinical variables related to pulmonary morbidity, including ppFEV_1_ and BMI. Additionally, it has also been associated with a reduced quality of life [8].

Health-related quality of life (HRQoL) is a multidimensional construct used as a health outcome and covers physical, psychological, and social functioning in daily life as seen from the patient's perspective [9]. Determinants of HRQoL in pwCF include lung function and BMI [10], treatment burden [11], lack of hope for the future [9], and sex. Consistent with sex differences regarding CS, female pwCF show lower HRQoL on several domains of generic and disease-specific scales, including global and mental health, than male pwCF [9]. Male and female pwCF perceive their health status differently, with females having a more accurate perception of their objective clinical health status. Additionally, women respond differently to poor health by reporting higher levels of physical or emotional symptoms than men [10].

Against this background, we report on explicitly captured CS in an adult cohort of pwCF and its relation to HRQoL, future health-related perspectives, and important clinical outcomes (ppFEV_1_ and BMI), also accounting for sex.

### *Hypotheses*

For the present study, we hypothesized that CS per se—by the above-outlined pathophysiology—and as an indicator of reduced therapy adherence in adults with CF results in lower ppFEV_1_ and BMI. As a result, adult pwCF with CS may have a reduced HRQoL and may be more concerned about the future. All these effects might be pronounced in women.

### *Hypotheses *_*1*__*A*_

CS correlates with lower ppFEV_1_.

### *Hypotheses *_*1*__*B*_

Females with CS show lower ppFEV_1_ than male patients with CS.

### *Hypotheses *_*2*__*A*_

CS coincides with lower BMI.

### *Hypotheses *_*2*__*B*_

Female patients with CS show lower BMI than male patients with CS.

### *Hypotheses *_*3*__*A*_

CS is accompanied by a lower QoL.

### *Hypotheses *_*3*__*B*_

Females with CS exhibit a lower QoL than males with CS.

### *Hypotheses *_*4*__*A*_

CS is associated with a less favorable perspective.

### *Hypotheses *_*4*__*B*_

Females with CS report a worse perspective of the future than males with CS.

### *Hypotheses *_*5*_

CS is an indicator of reduced therapy adherence.

## Methods

### Study design

**Fig. 1:**
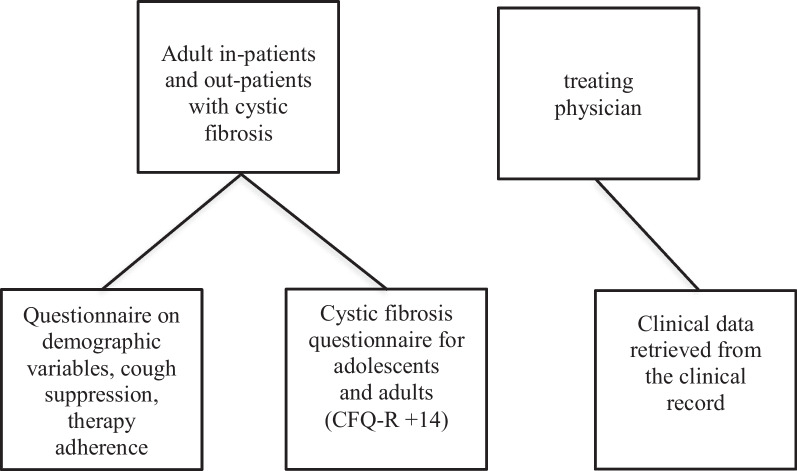
Study design

For the present cross-sectional questionnaire study conducted between January 2019 and March 2020, adult pwCF attending a CF specialist center were recruited. Each subject completed a questionnaire on CS/therapy adherence and HRQoL during a routine visit to the outpatient clinic or during an inpatient stay. Additionally, demographical and clinical data were assessed by the treating physician during the visit/stay (Fig. 1).

### Participants

All adult pwCF attending the CF center at Ruhrlandklinik, Universitätsmedizin Essen, Germany, were considered eligible for the study. The inclusion criteria were a confirmed diagnosis of CF according to the Cystic Fibrosis Foundation guidelines [12], an age of 18 years or older, and the ability to complete the questionnaires without help. Patients with CF after lung transplantation were excluded.

### Measures

#### Disease status

Objective measurements of disease status included ppFEV_1_, BMI, genotype, Pseudomonas aeruginosa colonization status, pancreatic function status, and the presence of CF-related diabetes mellitus.

#### Health-related Quality of Life

HRQoL was measured using the revised 'Cystic Fibrosis Questionnaire for adolescents and adults over 14 years old (CFQ-R + 14)', which has previously been validated and shows good psychometric properties for the German version for adolescents and adults with CF [13]. The CFQ-R + 14 measures daily functioning from the patient's perspective over the previous two weeks and consists of 50 items across the following 12 domains: physical functioning, role, vitality, emotional functioning, social, body image, eating disturbances, treatment burden, health perceptions, weight, respiratory symptoms, and digestive symptoms. Each domain is standardized on a 0–100 scale; higher scores indicate better HRQoL.

#### CS, therapy adherence, diagnosis disclosure

Data on CS, therapy adherence, and diagnosis disclosure were collected with a self-report questionnaire developed by the research team (Additional file 1: Table S1). The survey includes 12 items that measure participants' assessment of future health perspectives (one item), cough frequency and sputum quantity (two items), CS (one item), therapy adherence (three items), and diagnosis disclosure (four items, Additional file 1: Table S1). In addition, the participants were asked to specify their educational, employment, and marital status.

#### Statistical analyses

Data handling and statistical analyses were performed with SPSS 27.0 (IBM Corp., Armonk, NY). *H*_*1-4*_ were assessed by two-tailed testing, and the results were considered significant at *p* < 0.05. All other analyses were deemed exploratory, or the results were corrected for multiple comparisons controlling the two-tailed false-discovery-rate (FDR) at *q* < 0.05 [14] as indicated below. Effect size calculations relied on SPSS (semipartial correlation (*sr)—*small ≥ 0.1, medium ≥ 0.24, large ≥ 0.37) or an online calculator (*d—*small ≥ 0.2, medium ≥ 0.5, large ≥ 0.8) [15].

#### Correlation and multiple regression analysis

Categories of CS were collapsed into 3 categories (category 1: ‘never’ and ‘seldom’; category 2: ‘occasionally’; category 3: ‘frequently’ and ‘always’), and the 4 categories of sputum quantity were reclassified into 2 categories (category 1: ‘no’ and ‘little’; category 2: ‘moderate’ and ‘substantial’). The variable therapy adherence was coded by summing scores on the items self-administered physical therapy (none = 0, ≤ 1x/week = 1, 2-3x/week = 2; every day = 3), supervised physical therapy (none = 0, < 1x/week = 1, 1x/week = 2, > 1x/week = 3), and exercise (none = 0, < 1x/week = 1, 1x/week = 2, > 1x/week = 3) as a measure of adherence. Prior to analysis, all categorical variables were dummy coded.

Considering the sample size of the present study and a large number of potential covariates (age, genotype, cough frequency, sputum quantity, diabetes, exocrine pancreatic insufficiency, Pseudomonas colonization status, therapy adherence, employment status, marital status), an analysis-specific subset of covariates with a significant correlation with CS and the outcome measure of interest (ppFEV_1_, BMI, CFQ-R overall and subscale scores, or perspectives) was selected [16].

The linear relationship between ppFEV_1_, BMI, and CFQ-R (overall and subscale) scores (dependent variables) and CS (independent variable) was assessed by multiple linear regression considering covariates identified by the previous step of analysis. The interaction between CS and sex was modelled by multiplying both dummy-coded variables (2 dummy variables for CS, 1 dummy variable for sex). The results regarding the subscales of the CFQ-R were FDR-corrected for multiple comparisons. Considering the scale of measurement of the outcome perspectives, its relationship with CS was determined by ordinal logistic regression relying on a probit function, likewise accounting for important covariates.

Testing of the assumptions regarding the respective statistical procedure is detailed in the Additional file 1.

## Results

Of the 72 subjects with CF participating in the present study, 71 were considered for regression analysis considering the outcome measures ppFEV_1_ and BMI, and perspectives as a single case were excluded due to missing values on several of the covariates. Regarding the CFQ-R results, data from 63 participants were available for analysis. Participants who did not fully complete the CFQ-R did not differ from participants with complete data regarding demographic and clinical variables (Additional file 1: Tables S1 and S2).

### Demographics and clinical data

The subjects' demographic and clinical data are summarized in Table 1. Ninety-six percent of the pwCF who participated in the study were hospitalized. Almost half (48%) received hospital treatment because of pulmonary exacerbation, 20% started CFTR-modulator therapy, 11% took prophylactic antibiotic therapy, and 21% were hospitalized for miscellaneous causes. The mean age was 32.5 years (SD 11.68, range 18–71), and 57% of the sample was male. The average BMI was 20.27 kg/m^2^ (SD 3.42). Lung function indicated moderate disease severity (average ppFEV_1_: 43.17, SD 19.80). Most subjects disclosed their disease to family (100%) and friends (99%). Disclosure to employers and colleagues was shown by 58% of the subjects (Table 1). No sex differences were found.

**Table 1:** Patient demographic und clinical characteristics, n = 71

	All subjects (n = 71)	Male (n = 41)	Female (n = 30)
Age, years	32.52 ± 11.68 (18–71)	35.12 ± 12.64* (19–71)	28.97 ± 9.28 (18–52)
Sex		41 (58)	30 (42)
*Genotype n, (%)*			
*F508del homozygous*	28 (39)	12 (29)	16 (53)
*F508del heterozygous*	31 (44)	21 (51)	10 (33)
*Other*	12 (17)	8 (20)	4 (13)
BMI, kg/m^2^	20.27 ± 3.42 (15–33)	21.22 ± 3,68* (16–33)	19.97 ± 2.54 (15–25)
ppFEV_1_	43.17 ± 19.80 (16–99)	45.95 ± 21,26 (20–99)	39.37 ± 17.23 (16–82)
Pancreatic insufficiency n, %	46 (65)	22 (54)*	24 (80)
CF-related diabetes n, %	22 (31)	16 (39)	6 (20)
P. aeruginosa positive n, %	52 (73)	30 (73)	22 (73)
*Hospital n, %*			
*Inpatient*	68 (96)	39 (95)	29 (97)
*Outpatient*	3 (4)	2 (5)	1 (3)
Reasons for medical *treatment n, %*			
*PEX*	34 (48)	18 (44)	16 (53)
*Starting CFTR-modulator therapy*	14 (20)	11(27)	3 (10)
*IVAT*	8 (11)	4 (10)	4 (13)
*Others*	15 (21)	8 (20)	7 (23)
*Diagnosis disclosure n, %*			
Family	70 (99)	40 (98)	30 (100)
Friends	49 (70)	30 (73)	19 (66)
Employer	32 (78)	20 (83)	12 (71)
Colleagues	15 (37)	9 (38)	6 (35)

Results are presented as mean ± and standard deviation (SD) and range or number of pwCF n (%), ppFEV_1_—percent predicted forced expiratory volume in one second, PEX—pulmonary exacerbation; IVAT – intravenous antibiotic therapy (prophylactic). * Significant difference between sexes

### Bivariate correlations

Analyses revealed that CS was negatively correlated with the overall CFQ-R score (*r*(61) =  − 0.277, *p* = 0.005, *d* = 0.58) as well as most of its subscales except for the subscales eat, weight, and digestion (Additional file 1: Table S2), indicating that pwCF avoiding to cough experienced impaired HRQoL. In line with these findings, CS was also negatively correlated with perspectives (*r*(69) =  − 0.275, *p* = 0.01, *d* = 0.57), that is, deliberate CS was related to poor health-related prospects. Moreover, an exploratory correlation analysis between CS and sex (*r*(69) = 0.325, *p* = 0.004, *d* = 0.69) showed that CS was more frequent in females. There was also a statistical trend (0.05 < *p* < 0.10) of a negative relationship between CS and transformed ppFEV_1_ results (*r*(69) =  − 0.177, *p* = 0.06). However, CS did not correlate with BMI (*r*(69) =  − 0.07, *p* = 0.44).

### Multiple regression analysis

#### ppFEV_1_ (H_1A_) and BMI (H_2A_)

There was no significant relationship between CS and transformed ppFEV_1_ results (dummy 1 [never/rarely vs. occasionally]: *p* = 0.89; dummy 2 [never/rarely vs. often/always]: *p* = 0.34, Table 2; *H*_*1A*_ disproved) and transformed BMI (dummy 1 [never/rarely vs. occasionally]: *p* = 0.45; dummy 2 [never/rarely vs. often/always]: *p* = 0.54; *H*_*2A*_ disproved) considering important covariates either related to CS, the respective outcome measure assessed, or both.

**Table 2:** Regression Analysis

DV	IV	b	SE	*t*-value	*P*	sr
ppFEV_1_	BMI	1.37	0.65	2.12	0.04	0.22
	Cough suppression dummy 1	0.98	6.77	0.15	0.89	–
	Cough suppression dummy 2	8.95	9.23	0.97	0.34	–
	Future perspectives dummy 1	12.31	6.66	1.85	0.07	–
	Future perspectives dummy 2	20.36	7.82	2.61	0.01	0.27
	Cough suppresion x sex dummy 1	17.43	10.93	1.59	0.12	–
	Cough suppresion x sex dummy 2	− 0.55	11.80	− 0.05	0.96	–
BMI	ppFEV1	0.04	0.02	1.79	0.08	–
	Cough suppression dummy 1	0.93	1.23	0.76	0.45	–
	Cough suppression dummy 2	1.04	1.69	0.61	0.54	–
	Future perspectives dummy 1	0.30	1.25	0.24	0.81	–
	Future perspectives dummy 2	0.23	1.51	0.15	0.88	–
	Cough suppresion x sex dummy 1	− 1.12	2.04	− 0.55	0.59	–
	Cough suppresion x sex dummy 2	− 0.52	2.16	− 0.24	0.81	–
HRQoL—total score	ppFEV1	3.10	1.28	2.41	0.02	0.26
	Cough suppression dummy 1	− 74.68	72.12	− 1.04	0.31	–
	Cough suppression dummy 2	− 242.48	102.94	− 2.36	0.02	− 0.25
	Future perspectives dummy 1	− 43.53	72.88	− 0.60	0.55	–
	Future perspectives dummy 2	147.29	83.17	1.77	0.08	–
	Cough suppresion x sex dummy 1	72.55	116.17	0.62	0.54	–
	Cough suppresion x sex dummy 2	101.90	130.78	0.78	0.44	–
Future perspectives	ppFEV1	0.02	9 × 10–3	4.20	0.04	–
	Cough suppression dummy 1	0.05	0.44	0.01	0.92	–
	Cough suppression dummy 2	− 0.65	0.61	1.13	0.29	–
	Cough suppresion x sex dummy 1	1.38	0.80	2.98	0.09	–
	Cough suppresion x sex dummy 2	0.44	0.89	0.25	0.62	–

Regression results regarding the dependent variable (DV) outlined in the respective row. ppFEV_1_ = predicted forced expiratory volume in one second, HRQoL = health-related quality of life, b = unstandardized regression coefficient, SE = standard error, sr = semi-partial correlation (only reported regarding multiple regression results and significant findings). Cough suppression: dummy 1 [never/rarely vs. occasionally], dummy 2 [never/rarely vs. often/always]; future perspectives: dummy 1 [moderate vs. bad], dummy 2 [good vs. bad]; interaction between cough suppression and sex: dummy 1 [females vs. males—never/rarely vs. occasionally], dummy 2 [females vs. males—never/rarely vs. often/always]. Significant findings are printed in bold type. Note, ppFEV_1_ and BMI were transformed due to non-normality. Covariates adjusted for in addition to listed predictors: ppFEV_1_—sex, P. aeruginosa status, sputum quantity; BMI—age, sex, genotype (homo- vs. heterozygous), employment status; HRQoL—sex, ppFEV_1_; future perspectives—sex, sputum quantity, ppFEV_1_

#### HRQoL (H_3A_) and Perspectives (H_4A_)

More frequent CS was not associated with worse health-related perspectives (dummy 1 [never/rarely vs. occasionally]: *p* = 0.92; dummy 2 [never/rarely vs. often/always]: *p* = 0.29; *H*_*4A*_ disproved), but was significantly and negatively related to the overall score on the CFQ-R. Consequently, deliberate CS was related to impaired HRQoL (dummy 2 [never/rarely vs. often/always]: *p* = 0.02; *sr* =  − 0.251, *d* = 0.52; *H*_*3A*_ proved).

As revealed by exploratory subscale analysis of the CFQ-R, this finding was mainly driven by compromised daily physical functioning captured by the subscale ‘physical’ (dummy 2 [never/rarely vs. often/always]: *p*_*uncorrected*_ = 0.008, *p*_*FDR-corrected*_ = 0.24; *sr* =  − 0.263, *d* = 0.55). In line with this, there was a trend for a relationship between CS and the CFQ-R subscale ‘social’ (dummy 2 [never/rarely vs. often/always]: *p*_*uncorrected*_ = 0.03, *p*_*FDR-corrected*_ = 0.48; *sr* =  − 0.251, *d* = 0.52). Moreover, pwCF avoiding to cough also scored lower on the subscale ‘treatment burden’ (dummy 2 [never/rarely vs. often/always]: *p*_*uncorrected*_ = 0.01, *p*_*FDR-corrected*_ = 0.24; *sr* =  − 0.299, *d* = 0.63). However, none of these findings survived a correction for multiple comparisons.

#### Moderator [sex (H_1-4B_)] and mediator [(therapy adherence (H_5_)] analysis

In contrast to our hypotheses, there was neither an effect of sex on either outcome measure per se (*ppFEV*_*1*_: *p* = 0.33; *BMI*: *p* = 0.72; CFQ-R overall score: *p* = 0.46; perspective: 0.15) nor an interaction between sex and CS (*H*_*1-4B*_ disproved; s. Table 2). Moreover, there was no bivariate relationship between CS and therapy adherence (*r*(69) = 0.02, *p* = 0.83; *H*_*5*_ disproved), ruling out therapy adherence as a mediator.

## Discussion

With this cross-sectional study, we present for the first time that CS exists in adult pwCF, is more prevalent in women, and relates to reduced HRQoL. In contrast to previous studies in adolescents, which found that CS negatively correlated with ppFEV_1_ and BMI [6, 7], these findings could not be confirmed in adult pwCF. Furthermore, no relationship between CS and health-related perspectives of the future was observed. Despite the relationship between CS and HRQoL and a higher prevalence of CS in females, there was no significant sex difference regarding HRQoL or any other outcome measure, that is, ppFEV_1_, BMI, or perspectives.

The existence of CS in adult pwCF illustrates that this behavior persists into later stages of life. Eighty-five percent of the participating pwCF reported CS, of which 20% reported CS frequently and 6% reported CS consistently. Even in adulthood, women exhibit CS more frequently than men. In the present study, 98% of female subjects suppressed coughing, and 40% did so 'frequently' or 'always'. This finding is consistent with previous studies in adolescent pwCF, indicating that females of either age are more self-conscious about the impact of their coughing on others and show a tendency to suppress coughing to avoid negative public attention [6, 7]. From a social-cognitive perspective [17], CS undergoes stabilization as development progresses by reinforcement that may include avoiding negative public attention, thus avoiding cough-related negative emotions. Accordingly, in adults with CF avoiding cough, there was an uncorrected significant impairment of social life as assessed by the CFQ-R. Thus, negative emotions related to coughing may help preserve this behavior and negatively affect psychological well-being beyond adolescence.

Adolescent female patients with CS showed worse physiological outcomes [6, 7], but no such relationship was found in the adult cohort of pwCF in either sex regarding ppFEV_1_ and BMI in the present study. Thus, although CS is still more common in adult women with CF, the effects on physical outcomes are dissipating. As mentioned above, female adolescents appear to be more sensitive to cultural and social norms and are more concerned with socially conforming behavior. One possible explanation might be that female pwCF have become more self-accepting concerning their disease in adulthood. However, using disclosure of the CF diagnosis as a proxy for self-acceptance, there was no difference in this regard between female and male pwCF in the present study. Thus, the negative effect of CS on physical health could be offset by mechanisms other than those assessed in the present study in more detail. Moreover, these mechanisms might also partially be maladaptive, resulting in psychological distress. As mentioned above, there is suggestive evidence that adult female pwCF avoiding coughing may limit their social life to avoid the negative psychological impact of CS, which may also extend to other areas of life. However, this explanation warrants further well-powered studies addressing avoiding strategies in adult pwCF exhibiting CS.

In the present study, we did not find a relationship between CS and therapy adherence, a hypothesized mechanism linking CS to worse clinical outcomes as an indicator of non-adherence. Interestingly, despite the observation that subjects prone to CS are not less adherent, these subjects experienced a (uncorrected) significantly higher subjective treatment burden, again implying that CS may represent a proxy for increased psychological strain. Importantly, comparing findings regarding the relationship between CS and clinical outcomes with previous studies, it must be considered that formerly CS was operationalized by recording embarrassment about coughing and not CS itself, as in the present study.

Analysis of our data indicated that CS is associated with HRQoL. This effect was independent of physical health status investigated by lung function, BMI, or the presence of CFRD. This indicates that HRQoL in pwCF with CS cannot be explained by clinical indicators of disease status but instead seems to be associated with other variables at a subjectively experienced level. Since CF also influences psychological well-being, it is likely that mental health indicators have an impact on CS and possibly HRQoL. Unfortunately, this information as well as data on psychological morbidities, such as depressive symptoms, were not collected in the present observational study. Nonetheless, our results corroborate previous studies showing that quality of life is more strongly associated with mental health factors than physical health indicators [9, 18] and highlight the importance of screening patients’ mental health [8].

A weakness of the present study is the sample size and a potential bias through the inpatient testing procedure. Although the sample size was relatively large for a single-center CF study, it may not have been large enough to detect the complexities in the relationship of CS and the physiological and psychological outcomes. For example, the analyses regarding the relationship between CS and the CFQ-R + 14 subscales did not survive corrections for multiple comparisons.

Ninety-six percent of the participating subjects took part during an inpatient hospital stay and suffered from advanced disease status. Therefore, it was a relatively homogeneous group of participants. The inclusion of more outpatient pwCF experiencing different clinical conditions might have resulted in a more heterogeneous group; it would be quite interesting if this would have had an impact on the results. Nonetheless, this study is the first to examine CS regarding clinical parameters of disease severity and HRQoL in adult pwCF.

## Conclusions

This cross-sectional study addresses an important gap in clinical research regarding the relationship between explicitly assessed CS, clinical parameters, and HRQoL measured by a well-established questionnaire. As the only demographic variable, sex was related to CS. CS was significantly associated with HRQoL and showed no correlation with the studied physical parameters. It is likely that mental health indicators have an impact on CS. For further research, it is of particular interest to learn more about the mechanisms leading to CS and to devise practical targets for interventions to improve HRQoL in pwCF prone to CS.

## Supplementary Information

**Additional file 1:** Testing of the assumptions regarding the respective statistical procedure.

## Data Availability

The datasets used and/or analysed during the current study are available from the corresponding author on reasonable request.

## Abbreviations

CS: Cough suppression
CF: Cystic fibrosis
HRQoL: Health-related quality of life
ppFEV_1_: Percent predicted forced expiratory volume in one second
BMI: Body mass index
CFRD: CF-related diabetes
pwCF: People with cystic fibrosis
CFQ-R 14 +: Cystic fibrosis questionnaire for adolescents and adults over 14 years old
CFTR: Gene cystic fibrosis transmembrane conductance regulator gene
