# Supplementary material for: Cough suppression and HRQoL in adult people with cystic fibrosis: an unexplored correlation
Source: Health Qual Life Outcomes. 2022 Oct 6;20:141. doi: 10.1186/s12955-022-02053-2 (PMC9535958; doi:10.1186/s12955-022-02053-2)
Supplement: Supplementary file 1 — Additional file 1: Testing of the assumptions regarding the respective statistical procedure. [file 12955_2022_2053_MOESM1_ESM.docx]

**Additional File**

**Cough Suppression and HRQoL in Adults With CF: An Unexplored Correlation**

Ute Niehammer, Mathis Steindor, Svenja Straßburg MD, Sivagurunathan Sutharsan MD, Christian Taube MD, Matthias Welsner MD, Raphael Hirtz MD PhD, Florian Stehling MD

**Overview**

1. Supplementary Material and Methods - Evaluation of Statistical Assumptions
2. Supplementary Table S1 - Question and response choices of the self-report questionnaire to gain information about CS, therapy adherence, diagnosis disclosure
3. Supplementary Table S2 - Patient demographic und clinical characteristics regarding the results of CFQ-R +14, n = 63
4. Supplementary Table S3 - Bivariate Correlations
5. Supplementary Table S4 to Table S13 - Subscales CFQ-R + 14

- Table S4: Eat, Regression Results
- Table S5: social
- Table S6: treatment burden
- Table S7: physical
- Table S8: health perception
- Table S9: body
- Table S10: respiratory
- Table S11: role
- Table S12: emotion
- Table S13: weight
- Table S14: digestive
- Table S15: vitality

1. **Supplementary Materials – Evaluation of Statistical Assumptions**

|  |  |
| --- | --- |

Normality of the residuals of the multiple regression analyses regarding the outcome measures ppFEV_1_, BMI, and CFQ-R scores (dependent variables) were assessed by the Kolmogorov-Smirnov test. In case of non-normally distributed residuals, the dependent variable was rank-transformed according to (15), preserving their mean and standard deviation. If normality was not achieved by this approach, bootstrapping with 1000 bootstrap samples was performed. Multicollinearity was defined by variance inflation factors exceeding 10 and homoscedasticity was evaluated by the modified Breusch-Pagan test. Autocorrelations of residuals were excluded by the Durbin-Watson test, and outlier detection relied on Cook's distance (> 0.5).

Linearity of the relationship between continuous predictors and the logit transform of perspectives was assessed by the Box-Tidwell approach. As a goodness-of-fit measure relying on a χ2 statistic was employed, all pairs of categorial variables were inspected for sufficient cell size. The parallel lines test verified equivalent slope coefficients across the response categories of perspectives.

1. **Supplementary Table 1**

Question and response choices of the self-report questionnaire to gain information about CS, therapy adherence, diagnosis disclosure

| **Question** | **Response choices** |
| --- | --- |
| How do you assess your health perspectives in future? | Excellent / Moderate / Bad |
| How do you assess your frequency of coughing in clinically stable condition? | I'm coughing…  Not at all / Little / Moderate / a lot |
| Is there mucus when you are in clinically stable condition? | No / Yes, a little (one tablespoon) / Yes, moderate (one eggcup) / Yes, a lot (one cup) |
| When you are in public: Do you suppress coughing? | Never / Seldom / Occasionally / Frequently / Always |
| How frequent do you visit an assisted physical therapy? | Never / less than 1x/week / 1x/week / more than 1x/week |
| How frequent do you practice self-administered physical therapy? | Never / 1x/day / 2-3x/week / 1x/week / less than 1x/week |
| How frequent do you take your inhaled medication? | Never / 2-3x/day / 1x/day / several times a week / less than 1x/week |
| Do you exercise? | Yes / No |
| If so: How often do you exercise? | Less than 1x/week / 1x/week / more than 1x/week |
| Does your family know about your disease? | Yes / No/ Partly |
| Do your friends know about your disease? | Yes / No / Partly |
| Does your employer know about your disease? | Yes / No / Partly |
| Does your colleagues know about your disease? | Yes / No / Partly |

1. **Supplementary Table 2**

Patient demographic und clinical characteristics regarding the results of CFQ-R +14, n = 63

|  | all subjects (n=63) | male (n=37) | female (n=26) |
| --- | --- | --- | --- |
| Age, years | 32.98 ± 12.06  (18-71) | 35.46 ± 13.04  (19-71) | 29.46 ± 9.71  (18-52) |
| Sex |  | 37 (59) | 26 (41) |
| Genotype  n, (%)  *F508del homozygous*  *F508del heterozygous*  *Other* | 24 (38)  30 (48)  9 (14) | 10 (27)  20 (54)  7 (19) | 14 (54)  10 (38)  2 (8) |
| BMI, kg/m^2^ | 20.41 ± 3.45  (15-33) | 21.34 ± 3.72  (16-33) | 19.08 ± 2.56  (15-25) |
| ppFEV_1_ | 44.56 ± 20,24  (16-99) | 47.22 ± 21.61  (20-99) | 40.77 ± 17.82  (18-82) |
| Pancreatic insufficiency  n, % | 60 (95) | 34 (92) | 26 (100) |
| CF-related diabetes  n, % | 20 (32) | 14 (38) | 6 (23) |
| P. aeruginosa positive  n, % | 46 (73) | 27 (73) | 19 (73) |
| Hospital  n, %  *inpatient*  *outpatient* | 60 (95)  3 (5) | 35 (95)  2 (5) | 25 (96)  1 (4) |
| Reasons for medical treatment  n, %  *PEX*  *Starting CFTR-*  *Modulator Therapy*  *IVAT*  *Others* | 28 (44)  13 (21)  7 (11)  15 (24) | 16 (43)  10 (27)  3 (8)  8 (22) | 12 (46)  3 (12)  4 (15)  7 (27) |
| Diagnosis disclosure  n, %  family  friends  employer  colleagues | 62 (98)  43 (69)  30 (79)  14 (37) | 36 (97)  26 (70)  19 (83)  9 (39) | 26 (100)  17 (68)  11 (73)  5 (33) |

Results are presented as mean ± and standard deviation (SD) and range or number of patients n (%), ppFEV_1_ - percent predicted forced expiratory volume in one second, PEX - pulmonary exacerbation; IVAT – intravenous antibiotic therapy (prophylactic)

| 1. **Supplementary Table 3** Bivariate Correlations | | | | | |  |  |  |  |  |  |  |  |  |  |  |  |  |  |  |  |  |  |  |  |  |  |  |  |
| --- | --- | --- | --- | --- | --- | --- | --- | --- | --- | --- | --- | --- | --- | --- | --- | --- | --- | --- | --- | --- | --- | --- | --- | --- | --- | --- | --- | --- | --- |
|  | age | FEV1 | BMI | overall | physical | | vitality | emotion | eat | burden | health percep. | social | body | role | weight | respiratory | digestion | CS | future  perspec. | cough | genotype | adherence | sex | diabetes | PA | sputum | pancreas | work | familiy |
| age | 1,00 |  |  |  |  | |  |  |  |  |  |  |  |  |  |  |  |  |  |  |  |  |  |  |  |  |  |  |  |
| FEV1 | 0,04 | 1,00 |  |  |  | |  |  |  |  |  |  |  |  |  |  |  |  |  |  |  |  |  |  |  |  |  |  |  |
| BMI | 0,33^**^ | 0,28^*^ | 1,00 |  |  | |  |  |  |  |  |  |  |  |  |  |  |  |  |  |  |  |  |  |  |  |  |  |  |
| overall | -0,10 | 0,41^**^ | 0,12 | 1,00 |  | |  |  |  |  |  |  |  |  |  |  |  |  |  |  |  |  |  |  |  |  |  |  |  |
| physical | -0,08 | 0,58^**^ | 0,03 | 0,76^**^ | 1,00 | |  |  |  |  |  |  |  |  |  |  |  |  |  |  |  |  |  |  |  |  |  |  |  |
| vitality | -0,22 | 0,41^**^ | -0,02 | 0,85^**^ | 0,69^**^ | | 1,00 |  |  |  |  |  |  |  |  |  |  |  |  |  |  |  |  |  |  |  |  |  |  |
| emotion | -0,08 | 0,19 | -0,05 | 0,77^**^ | 0,43^**^ | | 0,63^**^ | 1,00 |  |  |  |  |  |  |  |  |  |  |  |  |  |  |  |  |  |  |  |  |  |
| eat | 0,15 | 0,15 | 0,29^*^ | 0,50^**^ | 0,31^**^ | | 0,28^*^ | 0,31^**^ | 1,00 |  |  |  |  |  |  |  |  |  |  |  |  |  |  |  |  |  |  |  |  |
| burden | -0,20 | 0,22 | -0,08 | 0,74^**^ | 0,51^**^ | | 0,63^**^ | 0,49^**^ | 0,23 | 1,00 |  |  |  |  |  |  |  |  |  |  |  |  |  |  |  |  |  |  |  |
| health perc. | -0,03 | 0,39^**^ | 0,02 | 0,77^**^ | 0,70^**^ | | 0,61^**^ | 0,61^**^ | 0,26^*^ | 0,51^**^ | 1,00 |  |  |  |  |  |  |  |  |  |  |  |  |  |  |  |  |  |  |
| social | -0,02 | 0,41^**^ | 0,04 | 0,77^**^ | 0,59^**^ | | 0,57^**^ | 0,68^**^ | 0,26^*^ | 0,45^**^ | 0,67^**^ | 1,00 |  |  |  |  |  |  |  |  |  |  |  |  |  |  |  |  |  |
| body | 0,11 | 0,30^*^ | 0,35^**^ | 0,64^**^ | 0,28^*^ | | 0,41^**^ | 0,54^**^ | 0,31^*^ | 0,37^**^ | 0,35^**^ | 0,52^**^ | 1,00 |  |  |  |  |  |  |  |  |  |  |  |  |  |  |  |  |
| role | -0,18 | 0,20 | -0,02 | 0,80^**^ | 0,61^**^ | | 0,72^**^ | 0,62^**^ | 0,28^*^ | 0,67^**^ | 0,61^**^ | 0,63^**^ | 0,44^**^ | 1,00 |  |  |  |  |  |  |  |  |  |  |  |  |  |  |  |
| weight | 0,00 | 0,20 | 0,37^**^ | 0,66^**^ | 0,29^*^ | | 0,42^**^ | 0,50^**^ | 0,43^**^ | 0,42^**^ | 0,37^**^ | 0,41^**^ | 0,45^**^ | 0,48^**^ | 1,00 |  |  |  |  |  |  |  |  |  |  |  |  |  |  |
| respiratory | 0,08 | 0,53^**^ | 0,16 | 0,74^**^ | 0,66^**^ | | 0,64^**^ | 0,35^**^ | 0,48^**^ | 0,50^**^ | 0,48^**^ | 0,48^**^ | 0,43^**^ | 0,49^**^ | 0,38^**^ | 1,00 |  |  |  |  |  |  |  |  |  |  |  |  |  |
| digestion | -0,12 | 0,08 | -0,07 | 0,35^**^ | 0,22 | | 0,31^*^ | 0,17 | 0,08 | 0,18 | 0,22 | 0,26^*^ | 0,16 | 0,16 | 0,15 | 0,31^**^ | 1,00 |  |  |  |  |  |  |  |  |  |  |  |  |
| CS | -0,10 | -0,18 | -0,07 | -0,28^**^ | -0,26^**^ | | -0,20^*^ | -0,28^**^ | -0,12 | -0,22^*^ | -0,23^*^ | -0,28^**^ | -0,27^**^ | -0,21^*^ | -0,05 | -0,20^*^ | -0,13 | 1,00 |  |  |  |  |  |  |  |  |  |  |  |
| future  perspec. | -0,11 | 0,35^**^ | 0,09 | 0,35^**^ | 0,35^**^ | | 0,41^**^ | 0,33^**^ | -0,03 | 0,34^**^ | 0,36^**^ | 0,29^**^ | 0,25^*^ | 0,28^**^ | 0,23^*^ | 0,32^**^ | 0,05 | -0,28^*^ | 1,00 |  |  |  |  |  |  |  |  |  |  |
| cough | -0,03 | -0,18 | -0,13 | -0,19 | -0,28^**^ | | -0,21^*^ | -0,15 | -0,24^*^ | -0,03 | -0,22^*^ | -0,13 | -0,02 | -0,15 | -0,02 | -0,16 | -0,06 | -0,12 | -0,19 | 1,00 |  |  |  |  |  |  |  |  |  |
| genotype | - | - | * | - | - | | - | - | - | - | - | - | - | - | - | - | - | 0,08 | 0,10 | 0,19 | 1,00 |  |  |  |  |  |  |  |  |
| adherence | 0,09 | -0,13 | 0,01 | 0,00 | 0,02 | | -0,02 | 0,07 | -0,07 | -0,20^*^ | 0,17 | 0,12 | 0,00 | 0,14 | 0,02 | -0,16 | 0,08 | 0,02 | 0,04 | 0,01 | 0,07 | 1,00 |  |  |  |  |  |  |  |
| sex | -,26^*^ | -0,15 | -0,33^**^ | -0,15 | -0,17 | | -0,04 | -0,10 | -0,14 | -0,10 | -0,19 | -0,17 | -0,21 | 0,00 | 0,04 | -0,25^*^ | -0,01 | 0,33^**^ | -0,09 | -0,05 | 0,24 | 0,11 | 1,00 |  |  |  |  |  |  |
| diabetes | 0,19 | 0,07 | 0,03 | -0,21 | 0,04 | | -0,09 | -0,17 | -0,13 | -0,11 | -0,12 | -0,25^*^ | -0,28^*^ | -0,13 | -0,01 | -0,20 | -0,16 | -0,09 | -0,15 | 0,12 | 0,04 | -0,02 | -0,20 | 1,00 |  |  |  |  |  |
| PA | 0,13 | -0,25^*^ | -0,11 | -0,24 | -0,22 | | -0,09 | -0,18 | -0,17 | -0,21 | -0,15 | -0,19 | -0,23 | -0,23 | -0,16 | -0,18 | 0,07 | 0,08 | 0,04 | -0,10 | 0,15 | 0,02 | 0,00 | 0,13 | 1,00 |  |  |  |  |
| sputum | 0,05 | -0,24^*^ | 0,07 | -0,25 | -0,28^**^ | | -0,15 | -0,07 | -0,05 | -0,27^*^ | -0,36^**^ | -0,10 | -0,12 | -0,09 | -0,06 | -0,32^**^ | -0,03 | 0,04 | -0,24^*^ | 0,36^**^ | 0,13 | 0,26^*^ | 0,05 | -0,02 | 0,03 | 1,00 |  |  |  |
| pancreas | -0,23 | -0,07 | -0,14 | 0,19 | -0,01 | | 0,12 | 0,14 | 0,08 | 0,18 | 0,12 | 0,28^*^ | 0,19 | 0,14 | -0,02 | 0,15 | 0,14 | 0,07 | 0,16 | -0,07 | 0,11 | 0,02 | 0,27^*^ | -0,91^**^ | -0,11 | 0,01 | 1,00 |  |  |
| work | 0,35^**^ | 0,16 | 0,27^*^ | 0,02 | 0,08 | | -0,06 | 0,01 | 0,00 | 0,00 | 0,15 | 0,07 | 0,09 | 0,01 | 0,04 | 0,20 | -0,05 | -0,05 | 0,10 | -0,2^*^ | 0,07 | -0,01 | -0,31^**^ | 0,00 | 0,24^*^ | -0,09 | -0,11 | 1,00 |  |
| family | 0,49^**^ | 0,04 | 0,15 | 0,14 | 0,08 | | 0,07 | 0,17 | 0,13 | -0,01 | -0,06 | 0,05 | 0,24^*^ | 0,05 | 0,13 | 0,08 | -0,05 | -0,15 | 0,12 | -0,15 | 0,21 | 0,11 | 0,01 | 0,07 | 0,09 | 0,16 | -0,10 | 0,27^*^ | 1,00 |
|  |  |  |  |  |  |  |  |  |  |  |  |  |  |  |  |  |  |  |  |  |  |  |  |  |  |  |  |  |  |

Bivariate correlations considering the scale of measure and potential violations of assumptions regarding the respective correlation measure. Note: Regarding the relationship between the categorical variable genotype and other continuous variables, multinominal logistic regressions have been performed. In these instances, only significant findings are flagged but no measure of association is reported as this would require a different table structure. * p < .05, ** p < .01. Abbreviations: burden – treatment burden, health percep. - health perception, cs – cough suppression, future perspec. – future perspectives, cough – cough frequency, PA – Pseudomonas aeruginosa, pancreas – pancreas insufficiency.

1. **Results of multiple regression regarding CFQ-R + 14 subscales**

Multiple regression results regarding the following CFQ-R + 14 subscales (Supplementary Tables 4-15). HRQoL = health-related quality of life, ppFEV_1_ = percent predicted forced expiratory volume in one second, b = unstandardized regression coefficient, SE = standard error, sr = semi-partial correlation CI = confidence interval. Cough suppression: dummy 1 [never/rarely vs. occasionally], dummy 2 [never/rarely vs. often/always]; future perspectives: dummy 1 [moderate vs. bad], dummy 2 [good vs. bad]; interaction between cough suppression and sex: dummy 1 [females vs. males - never/rarely vs. occasionally], dummy 2 [females vs. males - never/rarely vs. often/always]. Uncorrected significant findings are printed in bold type. Note: (1) the scores of the subscales weight, emotion, role, and digestion were transformed due to non-normality, (2) results reported regarding the subscale eat were bootstrapped.

**Supplementary Table 4**

Subscale CFQ-R + 14, HRQoL – Eat, Regression Results

|  | | | | |  | |  | |  | |  | |
| --- | --- | --- | --- | --- | --- | --- | --- | --- | --- | --- | --- | --- |
|  |  |  |  |  |  | |  | 95%-CI interval | | |  | |
|  | IV | b | SE | t-value | P | lower | | | | upper | |  |
|  | ppFEV_1_ | 0.18 | 0.22 | - | .36 | -0.25 | | | | 0.52 | |  |
|  | sex | -2.99 | 9.90 |  | .78 | -23.18 | | | | 18.92 | |  |
|  | Cough Suppression dummy 1 | -6.94 | 10.64 | - | .51 | -29.68 | | | | 15.29 | |  |
|  | Cough Suppression dummy 2 | -1.73 | 12.91 | - | .90 | -24.35 | | | | 25.13 | |  |
|  | Future Perspectives dummy 1 | -15.64 | 10.21 | - | .11 | -35.36 | | | | 4.98 | |  |
|  | Future Perspectives dummy 2 | -14.20 | 10.97 | - | .17 | -36.47 | | | | 8.49 | |  |
|  | Cough Suppression x Sex dummy 1 | 2.81 | 17.20 | - | .89 | -30.05 | | | | 37.82 | |  |
|  | Cough Suppression x Sex dummy 2 | -13.81 | 18.38 | - | .43 | -53.39 | | | | 20.02 | |  |

**Supplementary Table 5**

Subscale CFQ-R + 14, dependent variable: social

| IV | b | SE | t-value | P | sr |
| --- | --- | --- | --- | --- | --- |
| ppFEV_1_ | 0.25 | 0.14 | 1.80 | .08 | - |
| Sex | -13.18 | 8.56 | -1.54 | .13 | - |
| Cough Suppression  Dummy 1 | -1.69 | 7.68 | -0.22 | .83 | - |
| Cough Suppression  Dummy 2 | -23.85 | 10.96 | -2.18 | **.03** | -0.25 |
| Future perspectives  Dummy 1 | -0.52 | 7.76 | -0.07 | .95 | - |
| Future Perspectives  Dummy 2 | 8.89 | 8.85 | 1.00 | .32 | - |
| Cough Suppression x Sex  Dummy 1 | 16.70 | 12.38 | 1.35 | .18 | - |
| Cough Suppression x Sex  Dummy 2 | -26.76 | 13.92 | 1.92 | .06 | - |

**Supplementary Table 6**

Subscale CFQ-R + 14, dependent variable: treatment burden

| IV | b | SE | t-value | P | sr |
| --- | --- | --- | --- | --- | --- |
| ppFEV_1_ | 0.13 | 0.14 | 0.91 | .37 | - |
| Sex | -6.55 | 8.64 | -0.76 | .45 | - |
| Cough Suppression  Dummy 1 | -6.23 | 7.75 | -0.81 | .42 | - |
| Cough Suppression  Dummy 2 | -28.53 | 11.06 | -2.58 | **.01** | -0.30 |
| Future perspectives  Dummy 1 | 5.35 | 7.83 | 0.68 | .497 | - |
| Future Perspectives  Dummy 2 | 20.42 | 8.94 | 2.29 | **.03** | 0.27 |
| Cough Suppression x Sex  Dummy 1 | 3.24 | 12.49 | 0.26 | .80 | - |
| Cough Suppression x Sex  Dummy 2 | 24.21 | 14.05 | 1.72 | .09 | - |

**Supplementary Table 7**

Subscale CFQ-R + 14, dependent variable: physical

| IV | b | SE | t-value | P | sr |
| --- | --- | --- | --- | --- | --- |
| ppFEV_1_ | 0.79 | 0.15 | 5.14 | **<.001** | 0.49 |
| Sex | -8.27 | 9.60 | -0.86 | .39 | - |
| Cough Suppression  Dummy 1 | -8.45 | 8.61 | -0.14 | .33 | - |
| Cough Suppression  Dummy 2 | -34.06 | 12.29 | -0.49 | **.01** | -0.26 |
| Future perspectives  Dummy 1 | -0.04 | 8.70 | -0.001 | 1.00 | - |
| Future Perspectives  Dummy 2 | 13.57 | 9.93 | 0.21 | .18 | - |
| Cough Suppression x Sex  Dummy 1 | 1.64 | 13.88 | 0.02 | .91 | - |
| Cough Suppression x Sex  Dummy 2 | 21.03 | 15.61 | 0.25 | .18 | - |

**Supplementary Table 8**

Subscale CFQ-R + 14, dependent variable: health perception

| IV | b | SE | t-value | P | sr |
| --- | --- | --- | --- | --- | --- |
| ppFEV_1_ | 0.31 | 0.15 | 2.08 | .04 | - |
| Sex | 2.01 | 9.28 | 0.22 | .83 | - |
| Cough Suppression  Dummy 1 | -0.87 | 8.32 | -1.11 | .92 | - |
| Cough Suppression  Dummy 2 | -14.18 | 11.88 | -1.19 | .24 | - |
| Future perspectives  Dummy 1 | 7.61 | 8.41 | 0.90 | .37 | - |
| Future Perspectives  Dummy 2 | 25.63 | 9.60 | 2.67 | **.01** | 0.29 |
| Cough Suppression x Sex  Dummy 1 | -20.11 | 13.42 | -1.50 | .14 | - |
| Cough Suppression x Sex  Dummy 2 | -5.56 | 15.09 | -0.37 | .71 | - |

**Supplementary Table 9**

Subscale CFQ-R + 14, dependent variable: body

| IV | b | SE | t-value | P | sr |
| --- | --- | --- | --- | --- | --- |
| ppFEV_1_ | 0.15 | 0.18 | 0.81 | .43 | - |
| Sex | -9.69 | 11.36 | -0.85 | .40 | - |
| Cough Suppression  Dummy 1 | 0.31 | 10.19 | 0.03 | .98 | - |
| Cough Suppression  Dummy 2 | -13.39 | 14.54 | -0.92 | .36 | - |
| Future perspectives  Dummy 1 | -14.26 | 10.29 | -1.39 | .17 | - |
| Future Perspectives  Dummy 2 | 10.16 | 11.75 | 0.87 | .39 | - |
| Cough Suppression x Sex  Dummy 1 | 15.74 | 16.42 | 0.96 | .34 | - |
| Cough Suppression x Sex  Dummy 2 | -5.91 | 18.47 | -0.32 | .75 | - |

**Supplementary Table 10**

Subscale CFQ-R + 14, dependent variable: respiratory

| IV | b | SE | t-value | P | sr |
| --- | --- | --- | --- | --- | --- |
| ppFEV_1_ | 0.48 | 0.13 | 3.59 | **.001** | 0.37 |
| Sex | -17.82 | 8.41 | -2.12 | **.04** | -0.22 |
| Cough Suppression  Dummy 1 | -14.67 | 7.55 | -1.94 | .06 | - |
| Cough Suppression  Dummy 2 | -19.92 | 10.77 | -1.85 | .07 | - |
| Future perspectives  Dummy 1 | -4.35 | 7.63 | -0.57 | .57 | - |
| Future Perspectives  Dummy 2 | 11.63 | 8.70 | 1.34 | .19 | - |
| Cough Suppression x Sex  Dummy 1 | 18.25 | 12.17 | 1.50 | .14 | - |
| Cough Suppression x Sex  Dummy 2 | 9.93 | 13.68 | 0.73 | .47 | - |

**Supplementary Table 11**

Subscale CFQ-R + 14, dependent variable: role

| IV | b | SE | t-value | P | sr |
| --- | --- | --- | --- | --- | --- |
| ppFEV_1_ | 0.14 | 0.17 | 0.79 | .43 | - |
| Sex | -6.04 | 10.77 | -0.56 | .58 | - |
| Cough Suppression  Dummy 1 | -11.76 | 9.66 | -1.22 | .23 | - |
| Cough Suppression  Dummy 2 | -26.67 | 13.79 | -1.93 | .06 | - |
| Future perspectives  Dummy 1 | -0.87 | 9.77 | -0.09 | .93 | - |
| Future Perspectives  Dummy 2 | 14.76 | 11.14 | 1.33 | .19 | - |
| Cough Suppression x Sex  Dummy 1 | 19.39 | 15.58 | 1.25 | .22 | - |
| Cough Suppression x Sex  Dummy 2 | 13.20 | 17.52 | 0.75 | .45 | - |

**Supplementary Table 12**

Subscale CFQ-R + 14, dependent variable: emotion

| IV | b | SE | t-value | P | sr |
| --- | --- | --- | --- | --- | --- |
| ppFEV_1_ | 0.01 | 0.14 | 0.06 | .95 | - |
| Sex | -7.96 | 8.81 | -0.90 | .37 | - |
| Cough Suppression  Dummy 1 | 1.12 | 7.90 | 0.14 | .89 | - |
| Cough Suppression  Dummy 2 | -12.99 | 11.27 | -1.15 | .25 | - |
| Future perspectives  Dummy 1 | 2.04 | 7.98 | 0.26 | .80 | - |
| Future Perspectives  Dummy 2 | 19.72 | 9.11 | 2.17 | **.04** | 0.25 |
| Cough Suppression x Sex  Dummy 1 | 16.71 | 12.73 | 1.31 | .20 | - |
| Cough Suppression x Sex  Dummy 2 | 10.21 | 14.32 | 0.71 | .48 | - |

**Supplementary Table 13**

Subscale CFQ-R + 14, dependent variable: weight

| IV | b | SE | t-value | P | sr |
| --- | --- | --- | --- | --- | --- |
| ppFEV_1_ | 0.11 | 0.13 | 0.85 | .40 | - |
| Sex | 3.43 | 7.99 | 0.43 | .67 | - |
| Cough Suppression  Dummy 1 | -8.04 | 7.12 | -1.12 | .27 | - |
| Cough Suppression  Dummy 2 | -15.50 | 10.23 | -1.52 | .14 | - |
| Future perspectives  Dummy 1 | -3.31 | 7.24 | -0.46 | .65 | - |
| Future Perspectives  Dummy 2 | 11.16 | 8.26 | 1.35 | .18 | - |
| Cough Suppression x Sex  Dummy 1 | -1.93 | 11.55 | -0.17 | .87 | - |
| Cough Suppression x Sex  Dummy 2 | 9.14 | 12.99 | 0.70 | .49 | - |

**Supplementary Table 14**

Subscale CFQ-R + 14, dependent variable: digestive

| IV | b | SE | t-value | P | sr |
| --- | --- | --- | --- | --- | --- |
| ppFEV_1_ | 0.03 | 0.15 | 0.23 | .82 | - |
| Sex | 5.0 | 9.18 | 0.54 | .59 | - |
| Cough Suppression  Dummy 1 | -2.29 | 8.23 | -0.28 | .78 | - |
| Cough Suppression  Dummy 2 | -15.42 | 11.75 | -1.31 | .20 | - |
| Future perspectives  Dummy 1 | -15.84 | 8.32 | -1.90 | .06 | - |
| Future Perspectives  Dummy 2 | -4.94 | 9.49 | -0.52 | .61 | - |
| Cough Suppression x Sex  Dummy 1 | -2.73 | 13.27 | -0.21 | .84 | - |
| Cough Suppression x Sex  Dummy 2 | -2.72 | 14.93 | -0.18 | .86 | - |

**Supplementary Table 15**

Subscale CFQ-R + 14, dependent variable: vitality

| IV | b | SE | t-value | P | sr |
| --- | --- | --- | --- | --- | --- |
| ppFEV_1_ | 0.36 | 0.15 | 2.47 | **.02** | 0.26 |
| Sex | -4.88 | 9.09 | -0.54 | .59 | - |
| Cough Suppression  Dummy 1 | -7.76 | 8.15 | -0.95 | .35 | - |
| Cough Suppression  Dummy 2 | -18.68 | 11.63 | -1.61 | .11 | - |
| Future perspectives  Dummy 1 | 1.35 | 8.24 | 0.16 | .87 | - |
| Future Perspectives  Dummy 2 | 24.23 | 9.40 | 2.58 | **.01** | 0.27 |
| Cough Suppression x Sex  Dummy 1 | -18.68 | 13.14 | 1.28 | .21 | - |
| Cough Suppression x Sex  Dummy 2 | 16.79 | 14.78 | 0.95 | .35 | - |
